# Multiple Exon Skipping in the Duchenne Muscular Dystrophy Hot Spots: Prospects and Challenges

**DOI:** 10.3390/jpm8040041

**Published:** 2018-12-07

**Authors:** Yusuke Echigoya, Kenji Rowel Q. Lim, Akinori Nakamura, Toshifumi Yokota

**Affiliations:** 1 Laboratory of Biomedical Science, Department of Veterinary Medicine, Nihon University College of Bioresource Sciences, Fujisawa 252-0880, Japan; 2 Department of Medical Genetics, Faculty of Medicine and Dentistry, University of Alberta, Edmonton, AB T6G2H7, Canada; kenjirow@ualberta.ca (K.R.Q.L.); toshifum@ualberta.ca (T.Y.); 3 Third Department of Medicine, Shinshu University School of Medicine, Matsumoto 390-8621, Japan; anakamu@shinshu-u.ac.jp; 4 Department of Neurology, National Hospital Organization, Matsumoto Medical Center, Matsumoto 399-8701, Japan; 5 The Friends of Garrett Cumming Research and Muscular Dystrophy Canada HM Toupin Neurological Science Endowed Research Chair, Edmonton, AB, T6G2H7, Canada

**Keywords:** dystrophinopathy, Duchenne muscular dystrophy (DMD), Becker muscular dystrophy (BMD), dystrophin, hot spot, antisense oligonucleotide, multiple exon skipping, phosphorodiamidate morpholino oligomer (PMO or morpholino), exons 45–55 skipping, exons 3–9 skipping

## Abstract

Duchenne muscular dystrophy (DMD), a fatal X-linked recessive disorder, is caused mostly by frame-disrupting, out-of-frame deletions in the *dystrophin* (*DMD*) gene. Antisense oligonucleotide-mediated exon skipping is a promising therapy for DMD. Exon skipping aims to convert out-of-frame mRNA to in-frame mRNA and induce the production of internally-deleted dystrophin as seen in the less severe Becker muscular dystrophy. Currently, multiple exon skipping has gained special interest as a new therapeutic modality for this approach. Previous retrospective database studies represented a potential therapeutic application of multiple exon skipping. Since then, public DMD databases have become more useful with an increase in patient registration and advances in molecular diagnosis. Here, we provide an update on *DMD* genotype-phenotype associations using a global DMD database and further provide the rationale for multiple exon skipping development, particularly for exons 45–55 skipping and an emerging therapeutic concept, exons 3–9 skipping. Importantly, this review highlights the potential of multiple exon skipping for enabling the production of functionally-corrected dystrophin and for treating symptomatic patients not only with out-of-frame deletions but also those with in-frame deletions. We will also discuss prospects and challenges in multiple exon skipping therapy, referring to recent progress in antisense chemistry and design, as well as disease models.

## 1. Introduction

Dystrophinopathy comprises a spectrum of X-linked muscular dystrophies that are caused by mutations of the *dystrophin* (*DMD*) gene [1]. Of them, Duchenne muscular dystrophy (DMD) is a lethal neuromuscular disorder, which mostly arises from mutations that disrupt the translational reading frame and result in the lack of dystrophin, a muscle membrane-associated cytoskeletal protein that is critical for maintaining the stability and homeostasis [2]. The incidence of DMD is reported as being 10.7 to 27.8 per 100,000 live male births worldwide [3]. The clinical course of DMD is marked by the appearance of several severe symptoms. Duchenne muscular dystrophy patients suffer progressive and irreversible muscular damage, as is evident from them having dramatically elevated levels of serum creatine kinase (CK), followed by the development of proximal muscle weakness before the fifth year of age and then loss of ambulation by 12–15 years of age [4,5]. Cardiac and/or respiratory impairment frequently develop as a result of increased muscle weakness with age, leading to death by the 20–30 s [3,4]. Corticosteroid treatment is the current standard of care for the disease [6].

*DMD* mutations can also cause a less severe dystrophinopathy known as Becker muscular dystrophy (BMD), which has an incidence ranging from 5.8 to 7.2 in 100,000 live male births [7]. Unlike DMD, BMD is typically variable with mild, moderate to severe phenotypes; BMD patients are still capable of walking at least until their mid-teens or even until old age in some cases [8,9,10,11]. Although BMD does present clinical features such as hyperCKemia, calf hypertrophy and dilated cardiomyopathy (DCM) [4,12], these display widely variable severity among patients, ranging from those showing no symptoms at all to those who experience early death due to cardiac failure [12,13,14,15]. This variation of disease progression/severity is thought to relate to the different stabilities and expression levels shown by various truncated dystrophin proteins translated from reading frame patterns observed in BMD patients [16,17,18].

The phenotypic distinction between DMD and BMD is mostly explained by the reading frame rule. Severe DMD relates to frame-disrupting, out-of-frame mutations that do not allow dystrophin translation due to the creation of premature stop codons, whereas in-frame mutations are associated with mild BMD in which the reading frame is maintained for the production of internally-deleted, functional protein [19,20]. Deletions are the most frequent mutation type in dystrophinopathy, as they make up ~68% of all *DMD* mutations [21,22]. The reading frame rule holds true for more than 90% of DMD and BMD cases with out-of-frame and in-frame deletions, respectively [16,20,21,23]; exceptions are generally explained by the transcriptional status of the gene [24,25,26,27]. Therefore, disease severity can largely be determined by the frameshift types of defective *DMD* gene variants and resulting dystrophin isoforms.

This genotype-phenotype association underlies the concept of exon skipping therapy using single-stranded nucleic acid analogs called antisense oligonucleotides (AOs). Synthetic AOs bind to corresponding sites in precursor mRNAs (pre-mRNAs) in a sequence-specific manner and exclude frame-disrupting exons during RNA splicing. Taking advantage of this nature, AO-mediated exon skipping aims to convert out-of-frame mRNAs to in-frame ones, so that shortened yet partially functional dystrophin can be produced in DMD patient muscles and help delay disease progression. Exon skipping using AOs designed against particular exons can theoretically be applied to a variety of *DMD* mutations.

Twenty years since the demonstration of its first success in skipping a *DMD* exon in human cells [28], AO-mediated exon skipping is nearing meaningful clinical application for the treatment of DMD. In 2016, the US Food and Drug Association (FDA) has approved the first-ever *DMD* antisense drug for exon 51 skipping, eteplirsen (Exondys51) [29]. Clinical trials for AOs targeting exons 45 and 53 for skipping have also been undertaken [30,31]. Single exon skipping strategies, however, currently face a couple of major challenges. These include that the applicability is limited mostly to patients with out-of-frame mutations and that various dystrophin isoforms with different functionality are produced in this approach, depending on patient mutation patterns. In DMD patients with deletion mutations, that are the most common mutation type, while single exon skipping can cover approx. 70% of these patients, especially those having deletions in the rod domain [32], multiple exon skipping can theoretically be applied to approx. 90% of this population [33]. Also, dystrophin structures generated by single exon skipping vary greatly depending on patient mutation patterns, raising a concern whether AO therapy can provide its expected benefit equally across patients, for example, whether the therapeutic effect an exon 51 skipping AO has on a patient with an exon 52 deletion can be observed similarly on a patient with an exon 45–50 deletion. In contrast, multiple exon skipping is supposed to produce a consistent form of dystrophin with a particular structure and function regardless of patient mutation patterns, ensuring that similar levels of functionality of the restored dystrophin protein are observed among different treated patients. Multiple exon skipping using combinational AOs in a cocktail formulation is therefore expected to become the new therapeutic modality that expands the potential of the exon skipping approach.

To develop effective multiple exon skipping strategies, the therapeutic potential and general versatility to patients need to be thoroughly assessed from *DMD* genotype-phenotype associations. For this purpose, global/nationwide DMD databases that coordinate data of genetic and clinical diagnoses have been established [21,22,34,35,36]. These databases have gotten more useful and reliable with increases in patient registration as well as the advancement of molecular diagnostic methods. The amount of clinical evidence describing the phenotypes of patients with specific exon deletions has also increased, including exceptional cases in which specific exon deletions are associated with mild symptoms or asymptomatic courses: that is, the deletions of exons 45–55 [8,9,10,11,37,38,39] and exons 3–9 [40,41]. Both DMD databases and clinical reports provide valuable information that underlie the development of multiple exon skipping strategies.

In this review, utilizing the Leiden DMD database that currently contains more than 16,000 patient registrations, we update the spectrum of deletion mutations and their corresponding phenotypes to provide a theoretical rationale for the development of multiple exon skipping therapies, in particular exons 45–55 skipping [37,42] and exons 3–9 skipping [40,41]. Importantly, this review highlights the need for treating symptomatic patients not only with out-of-frame deletions but also those with in-frame deletions, as well as the need for rationally designing exon skipping strategies such that they lead to the production of truncated dystrophin proteins with preserved functionality. In addition, we will discuss the challenges multiple exon skipping currently faces in its development, focusing on the chemistry and design of cocktail AOs and the humanized disease models that have been used to test this strategy thus far.

## 2. Mutational Hot Spots and Genotype-Phenotype Associations in the *DMD* Gene

The *DMD* gene is the largest gene in humans which spans 2.4 million bases and consists of 79 exons. A variety of mutations occur throughout the gene: such as deletion, duplication and point mutations that include splice site mutations, nonsense mutations and so forth. Among them, large deletion mutations (≥1 exon) are the most common type and account for 68% of all *DMD* mutations [22]. Although large, mutations do not arise in the gene at random. In the previous large cohort using over 7000 patient data, two regions ranging from exon 2 to 20 and exon 45 to 55 have been reported as hot spots for *DMD* deletions [22].

Owing to massive efforts of data collection over a few decades, public and reliable DMD mutation databases are now easily accessible such as the UMD-DMD France database [21], TREAT-NMD DMD Global database [22] and the Leiden Open Variation Database (LOVD) [20,43] in which the number of patients has reached 2898, 7150 and 16,106, respectively (accessed 13 October 2018). Of them, the LOVD involves more detailed information regarding patient data, in particular, the molecular diagnostic methods used. Currently, several reliable diagnostic techniques are available to accurately identify deletion patterns: for example, Multiplex Ligation-dependent Probe Amplification (MLPA), Multiplex Amplifiable Probe Hybridization (MAPH), Array Comparative Genomic Hybridization (array CGH), Next Generation Sequencing (NGS), or a combination of multiplex PCR and Southern blotting [44,45,46]. In this context, the previous analyses utilizing over 4700 patients have given significant information for developing exon skipping therapies [20,32]. Since the previous reports, the number of patients registered in the LOVD v.3.0 has increased to more than 16,000, enough to update the spectrum of *DMD* mutations and phenotypes. For a more in-depth assessment of the therapeutic potential of the exon skipping approach, we therefore updated information on the mutation frequency and hot spots of large exonic deletions. Importantly, we analyzed only data of genotypes determined by MLPA and equivalents, as described above, and consequent phenotypes.

**Figure 1:**
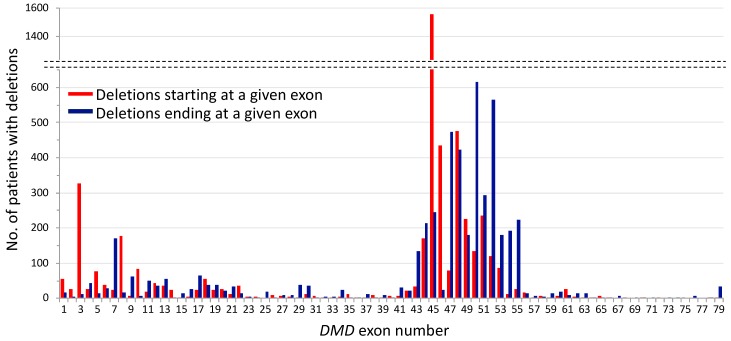
*DMD* hot spots of deletion mutations in the proximal (exons 1–22) and distal (exons 43–55) regions. The two hot spots were found using data from 4929 patients in the LOVD database (as of 22 June 2018) who harbor deletions starting or ending at a given exon; for example, the deletions of exons 45–XX such as exons 45–50 and 45–52 fall into the group of “deletions starting at a given exon” shown in red; exons XX–50 deletions such as exons 48–50 and 49–50 deletions come into the group of “deletions ending at a given exon” in blue. Individual deletion patterns found in the proximal and distal hot spots account for 0.5–6.6% and 2.7–12.5% of all the deletions, respectively; others account for less than 0.5%, except deletions involving exons 29, 30, 41, 61 and 79. Only deletions identified by Multiplex Ligation-dependent Probe Amplification (MLPA), Multiplex Amplifiable Probe Hybridization (MAPH), Array Comparative Genomic Hybridization (array CGH), Next Generation Sequencing (NGS), or a combination of multiplex PCR and Southern blotting were analyzed.

Here, using acceptable data from 4929 subjects with deletions, we confirmed two hot spots in the *DMD* gene: exons 1–22 in the proximal region and exons 43–55 in the distal region (Figure 1). In the proximal hot spot, 23% of all the deletions started from an exon in the exons 1–22 region and 16% ended at an exon in this region. Deletions having its first and last exon within the distal hot spot made up 73% and 76% of the total deletions, respectively. Accordingly, 96% and 92% of the deletions started from and ended at an exon allocated in the hot spots, respectively. Consistent with the previous large cohort [21], the analysis confirmed that the most common deletion pattern is exons 45–XX deletions with an enormous number of 1554 patients having this set of mutations, followed by the deletions of exons XX–50 and exons XX–52, which includes more than 500 patients. It was clearly shown that among exons within the hot spots, these three exons, particularly exon 45, have a higher susceptibility to deletion-generating genomic rearrangements. Although largely unknown, frequent deletions of particular exons may be partially explained by the existence of more breakpoints in the flanking introns of these exons [47,48,49].

**Figure 2:**
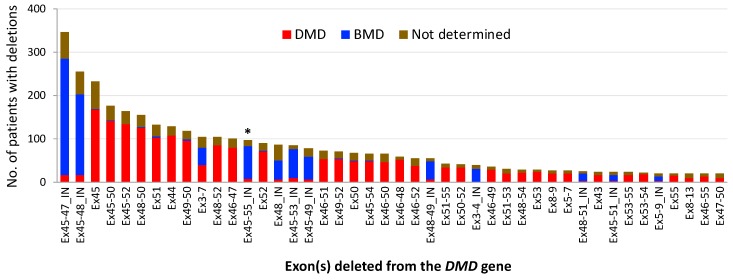
The frequency of large deletion mutations (≥1 exon) and its phenotypic spectrum. A total of 4929 patients with deletions from the LOVD database (as of 22 June 2018) were used. Deletions involving 20 or more patients are shown. Note the top two mutations are in-frame deletions and exons 45–55 deletion is ranked at 13th. Asterisk, exons 45–55 deletion; “IN,” in-frame deletions (otherwise out-of-frame deletions). DMD: Duchenne muscular dystrophy; BMD: Becker muscular dystrophy.

In the analysis, we found as many as 538 different patterns of large deletions in total, which included 283 and 213 patterns of out-of- and in-frame deletions, respectively; the remaining 42 deletions were ones with exon 1 and/or 79 that do not fall within the frameshift category. Of them, 44 deletion patterns consisted of 20 or more patients, as shown in Figure 2: 38 from the distal hot spot and 6 from the proximal hot spot. The represented deletions accounted for 71% of all the deletions analyzed. The first twelve deletion patterns had more than 100 patients each and occur in 2% to 7% of deletions (mostly in the distal hot spot; exons 3–7 deletion was the only one from the proximal hot spot). The top five were deletions starting at exon 45. The first and second in the ranking were in-frame deletions of exons 45–47 and 45–48, which involved more than 300 and 200 patients, respectively. Importantly, exons 45–55 deletion was placed third in the ranking of in-frame deletions and thirteenth of all the deletion patterns, indicating that this large deletion with eleven exons is defined as one of the most common deletions (2% of the total, 98 out of 4929).

Consistent with previous reports [21,22], mutational spectrum analysis confirmed that exon 45 deletion is the most common out-of-frame deletion, involving 232 patients that account for approx. 5% of the total deletions. This deletion is amenable to exon 44 or 46 skipping. However, there is little information as to the clinical presentations that underlie the skipping strategies. Patients harboring the in-frame deletion of exons 44–45, as observed following exon 44 skipping, have never been reported in any DMD databases at this time. In contrast, five patients with in-frame exons 45–46 deletion have been registered as DMD (80%, 4 in 5) in the LOVD using MLPA or equivalent methods. A clinical study also has shown that this deletion is more associated with DMD (4 out of 4 patients) [50]. The findings suggest that the exons 45–46 deleted-truncated dystrophin is potentially unstable and that multiple exon skipping, such as exons 45–55 skipping, can become an alternative option to be applied to patients with an exon 45 deletion.

**Figure 3:**
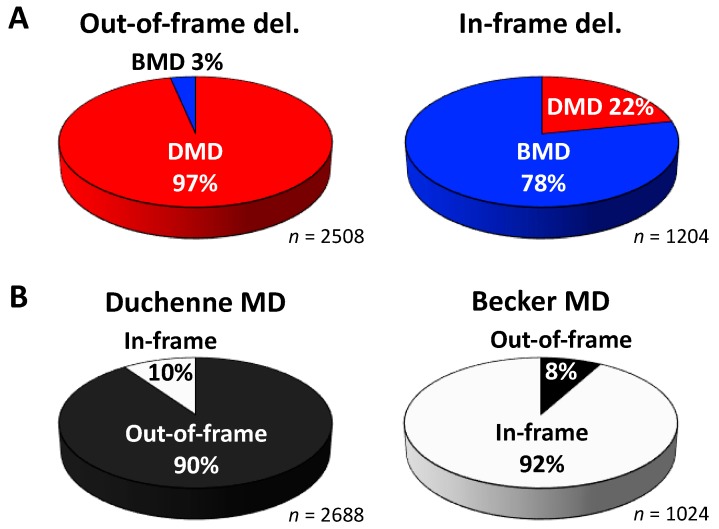
Proportions of phenotypes and frame types in deletion (del.) mutations. The analysis was performed using a total of 3712 patients having deletions arising within the region of exons 2–78 to which the definition of frameshift: out-of- or in-frame is applied. Patients without diagnosis with DMD or BMD were excluded from the analysis. (**A**) “Frame type-based” phenotype ratios indicating that 22% of patients having in-frame deletions are associated with DMD. (**B**) “Phenotype-based” frame type ratios that define the reading frame rule [19,20].

Associated with the above representative findings, the frame type-based phenotypic analysis here revealed 97% DMD cases in out-of-frame deletions (2508) and surprisingly, 22% DMD cases in in-frame deletions (1204) (Figure 3A). It should be noted that Figure 3 represents the proportion of two phenotypes in the out-of- or in-frame deletion type. Unlike the phenotypic analysis, the analysis using the reading frame rule which refers to the proportion of two frame types in DMD or BMD [19], namely phenotype-based frame type analysis, held true for 90% out-of-frame deletions in 2688 DMD patients and 92% in-frame deletions in 1024 BMD patients (Figure 3B). Together, the genotype-phenotype associations analyzed here further underscored that in-frame deletions do not always result in the milder BMD and that single exon skipping, hence, may not necessarily lead to a favorable therapeutic outcome. This implies the need for converting DMD-associated in-frame mRNAs to particular, potentially functional in-frame ones. Accordingly, the present findings underlie the development of multiple exon skipping therapy, in particular, exons 45–55 and exons 3–9 skipping, which aim for the functional correction of dystrophin. Such multiple exon skipping could become a promising therapeutic approach for DMD patients caused by out-of- and in-frame deletions in the *DMD* hot spots.

## 3. Exons 45–55 Skipping: Rationale and Pre-Clinical Tests

**Figure 4:**
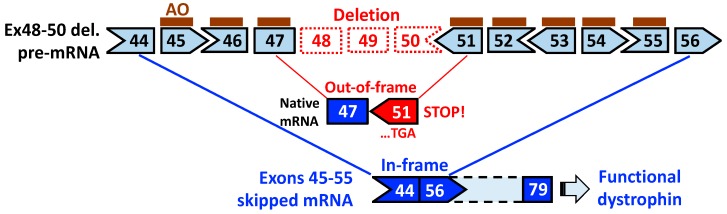
Schematic of exons 45–55 skipping using cocktail antisense oligonucleotides (AOs) for *DMD* exons 48–50 deletion. Individual AOs are designed to skip each exon within the region from exon 45 to 55. Native out-of-frame mRNAs cannot be translated into dystrophin due to a newly generated premature stop codon in exon 51. Skipping the entire exons 45–55 region by treatment of an AO cocktail leads to the production of truncated but functional dystrophin.

Currently, it is widely recognized that the in-frame mutation of *DMD* exons 45–55 deletion represents mild symptoms or an asymptomatic course of disease [8,9,10,11,37]. In 1991, exons 45–55 deletion was first found in a male patient at the age of 15 years who showed only high serum CK levels and no muscle symptoms [51]. Since then, three patients (the age of 26, 36 and 69 years at the diagnosis) have been diagnosed with the deletion of exons 45–55 and their detailed clinical status were followed up for a decade or more by Nakamura and colleagues [11,52,53]. They confirmed that this deletion led to no obvious skeletal muscle involvement in this observation period, except persistently high serum CK levels (580–1347 IU/L) and cardiac involvement that were controllable with pharmaceutical agents. Together with these clinical observations, a surprising finding is that some BMD patients with exons 45–55 deletion, which were molecularly diagnosed by MLPA or a combination of multiplex PCR and Southern blotting, retained ambulation until the late age of 60s–70s [8,9,10,11,37,38]. In the current LOVD, it is shown that approx. 90% of patients holding exons 45–55 deletion, which is registered by MLPA or equivalents, are BMD patients (75 out of 83 patients having the diagnosis with DMD or BMD). Contrary to the general notion that BMD patients exhibit heterogeneous phenotypes even if the same *DMD* mutation is present [9,13,15,39], the consistently mild or subclinical phenotype found in this particular deletion provides a theoretical rationale for the development of exons 45–55 skipping therapy (Figure 4).

**Figure 5:**
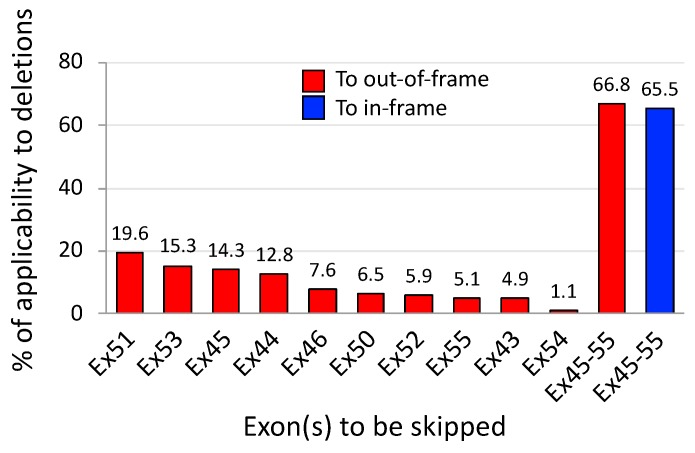
Theoretical applicability of exons 45–55 skipping to patients with out-of- and in-frame deletions. 3232 and 1611 patients with out-of-frame and in-frame deletions, respectively, were extracted from the Leiden DMD database (as of 22 June 2018). The applicability to out-of- (red) and in-frame deletions (blue) was examined. As single exon skipping has not been fully correlated with functional correction, the applicability only to out-of-frame deletions is shown. In contrast, the applicability of exons 45–55 skipping thought to enable the production of functionally improved dystrophin, is shown for both frame types.

In 2007, the concept of exons 45–55 skipping was proposed by Béroud et al. [37]. They used the UMD-DMD database and predicted the theoretical applicability of this therapeutic strategy to be at 63% of DMD patients with deletions (161 out of 254) and 42% of all 602 DMD patients regardless of mutation type. Similar to this study, Figure 5 from our analysis also revealed that exons 45–55 skipping therapy was applicable to approx. 67% of out-of-frame deletions (2159 out of 3232); note out-of-frame deletions are related to a DMD phenotype at 97% of cases (Figure 3A). In contrast, the applicability of single exon skipping targeting an exon within exons 45 to 55 was predicted to range from 1% to 20% of cases in this frame-disrupting type. The present analysis also revealed that exons 45–55 skipping can be applied to approx. 66% of in-frame deletions (1056 out of 1611); note in-frame deletions are associated with DMD at 22% of cases (Figure 3A). Furthermore, exons 45–55 skipping is applicable to approx. 66% of all deletions analyzed here (3215 out of 4929; both frame types in the total deletions that include exons 1- and 79-related deletions); 44% of all out-of-frame deletions, 2159 out of 4929; and 21% of all in-frame deletions, 1056 out of 4929.

Given the exceptionally milder symptoms or asymptomatic course observed with this particular deletion, as well as its expanded applicability in patients, this indicates that exons 45–55 skipping has the potential for becoming a therapeutic option for symptomatic patients with DMD, BMD and intermediates, caused by both in-frame deletions as well as out-of-frame deletions. The need for such an approach is further underlined by the fact that many BMD patients develop cardiomyopathy which is a leading cause of death, in spite of their mild or moderate course of skeletal muscle involvement [54]. The pathogenesis remains unclear but it is likely to be associated with an imbalance in blood-pumping function between skeletal and cardiac muscles that forces an overload to the heart [55]. Functionally corrected dystrophins produced by multiple exon skipping such as exons 45–55 skipping may help prevent the development of cardiomyopathy in BMD, as associated with improved muscle functions expected upon treatment.

With its expected therapeutic advantage, exons 45–55 skipping was first empirically tested using cocktail AOs with the 2′-*O*-methyl-phosphorothioates (2′-*O*MePS) chemistry in primary DMD muscle cells having exons 48–50 deletion, but no substantial amount of exons 45–55 skipped mRNA was found [56]. Four years since the first attempt, Aoki et al. demonstrated the proof-of-concept of exons 45–55, skipping using a cocktail of 10 AOs which were modified with the vivo-phosphorodiamidate morpholino oligomer (vivo-PMO) chemistry, in a mouse model called *mdx52* with an exon 52 deletion in the mouse *Dmd* gene [42]. A subsequent study revealed the long-term efficacy and safety of exons 45–55 skipping with the 10-vivo-PMO cocktail after systemic treatment of the mouse model [57]. A series of studies have confirmed the ability of an AO cocktail to simultaneously skip 10 exons of the maximum number in a deletion amenable to exons 45–55 skipping. The cocktail AO strategy conducted in the series can be applied with different combinational AOs to other deletions. Encouragingly, we have recently shown the successful skipping of the entire exons 45–55 region in the human *DMD* gene, where DMD patient-derived myotubes having a deletion of either exons 45–50 and 46–50 were treated with five- and six-PMO cocktails, respectively [58]. Besides the cocktail approach, gene editing using CRISPR/Cas9 has also proven efficacious in deleting the entire *DMD* exons 45–55 region from the genome, accompanied by dystrophin rescue, in DMD patient-derived induced pluripotent cells (DMD iPSCs) [59] and a humanized DMD mouse model [60]. The attempts of skipping and deleting exons 45–55 so far to data are summarized in Table 1.

## 4. Expression Levels and Function of Exons 45–55-Deleted Dystrophin

In DMD therapy, it is controversial how much rescued dystrophin is required to lead to therapeutic benefit. This issue has been made more apparent in the process of eteplirsen approval by the FDA [62]. In this review process, an idea was advocated that having between undetectable and 10% of normal dystrophin levels, as represented by Western blotting, is reasonably likely to predict clinical benefit. This interpretation is somewhat reasonable with the given explanations [62] but there is little doubt as to the notion that higher expression of dystrophin can lead to a better therapeutic outcome. Therapeutically beneficial levels of dystrophin seem to vary depending on mutation status and other factors that modify phenotypes in individuals. Indeed, the various protein levels, as represented by Western blotting, have been reported among different mutation types/patterns in BMD patients and have been associated with their severity: for example, less than 10% of normal levels in severe BMD patients and no correlation of the levels (13% to 76%) with the severity found in exons 45–47 deletion [17], the range from 29% to 57% in patients with 5’ proximal intronic/exonic mutations who have cardiac involvement but no obvious muscle weakness [63] and >40% levels in mild or asymptomatic patients with deletions within the distal hot spot [9]. In terms of the exons 45–55 deletion, although the numbers available are limited, patients with this deletion were reported to yield relatively higher dystrophin amounts (50 to 100% of normal) than those with other in-frame deletions [9,64,65,66]. These observations pose a question as to how the milder phenotype found with this particular deletion is associated with the levels of dystrophin expression in patients. The efficacy of exon skipping can be determined primarily with examining the structure and expression levels of rescued dystrophin. To provide more rationale to exon 45–55 skipping therapy, a comprehensive assessment of associations among three variables in patients, namely severity and dystrophin expression levels and function, needs to be done.

Associated with the above, the function of exons 45–55-deleted truncated dystrophin that underlies its clinical benefit remains unclear. In response to this, Tanihata et al. recently created a new humanized *mdx* mouse model that harbors the exons 45–55-deleted human *DMD* gene but does not express mouse *Dmd*-derived dystrophin due to a *Dmd* mutation [67]. This novel transgenic mouse model was confirmed to exhibit a mild phenotype comparable with wild-type mice in terms of CK levels, sarcolemma stability, pathology, muscle contractile force and levels of dystrophin expression in muscles. The activity of neuronal nitric oxide synthase (nNOS), of which its binding site in dystrophin is affected due to the deletion of exons 45–55, was recovered in the transgenic mice to the levels found in wild-type mice, despite the mis-localization of nNOS to the cytoplasm. It was also suggested that the truncated dystrophin normalizes Ca^2+^ uptake into the sarcoplasmic reticulum through the increased activity of sarcoplasmic/endoplasmic reticulum Ca^2+^-ATPase (SERCA) and the reduction in expression levels of a SERCA-inhibitory peptide, sarcolipin. The study revealed that the functionality of dystrophin lacking the exons 45–55-encoded region is nearly equivalent to that of full-length dystrophin. Although the findings further support the development of exons 45–55 skipping for clinical application, it remains unclear whether the mild phenotype found in this model is associated with the high expression levels of the rescued dystrophin lacking exons 45–55 region. It needs to be experimentally clarified how the exons 45–55-deleted dystrophin works better compared to other internally-truncated dystrophins.

## 5. Exons 3–9 Skipping: Rationale and Challenges

Patients with mutations in the 5’ proximal hot spot have been reported to exhibit variable phenotypes, ranging from an asymptomatic course to lethal DMD depending on mutation patterns and/or unknown factors that could either aggravate or ameliorate symptoms [21,68,69]. A typical example to explain this variation is cases with the out-of-frame deletion of exons 3–7. This deletion is supposed to cause DMD but the UMD-DMD database shows that more than half of the patients have been diagnosed with BMD (59%, 19 out of 32 patients) [21]. The exception was also confirmed in the present analysis with data on deletions that are determined by MLPA or equivalent techniques: 50% of 80 cases with an exons 3–7 deletion had BMD (Figure 2). This case can be partially explained with the presence of newly generated start codons [70,71] and some genetic modifiers in patients [72]. As an opposite case, a patient with an in-frame exon 5 deletion has been reported to develop an unexpectedly moderate/severe BMD phenotype [73]. In the Leiden DMD database, 5 patients with this in-frame deletion, which is expected to exhibit the BMD phenotype, have been enrolled and one of them has been diagnosed with DMD (others to be determined). The variable phenotypes are usually thought to be associated with the impact of mutations on the actin-binding domain 1 (ABD1) that is encoded by exons 2–8 and which essentially acts to stabilize muscle membrane [68,69,74]. However, this theory may not be enough to explain both cases described above. An implication from these cases is that particular combinations of skipped exons may serve clinical benefits in the proximal hot spot, as supported by the in-frame deletion of exons 3–9 deletion [40,41,75].

**Figure 6:**
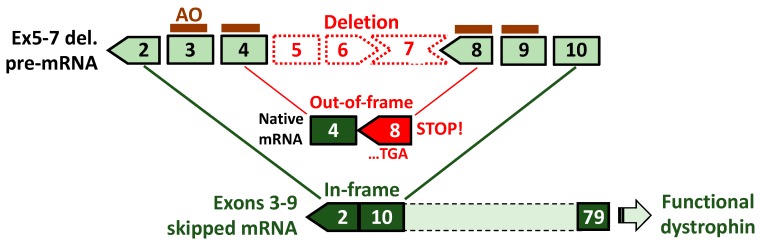
Schematic of exons 3–9 skipping using cocktail antisense oligonucleotides (AOs) for *DMD* exons 5–7 deletion. Individual AOs are designed to skip each exon within the region of exons 3–9. Native out-of-frame mRNAs do not allow for the production of dystrophin due to a generated premature stop codon in exon 8. Skipping the entire exons 3–9 region by cocktail AOs leads to the expression of truncated but partially functional dystrophin.

In this context, Nakamura et al. have recently proposed a multiple exon skipping therapy targeting ABD1-coding exons, i.e., exons 3–9 skipping (Figure 6) based on the clinical evidence of a patient with an in-frame exons 3–9 deletion who followed an asymptomatic course over a decade or more from 15 years of age [40]. The patient, whose mutation was diagnosed using MLPA, exhibited none of the typical dystrophic symptoms, including muscular weakness and atrophy, easy fatigability, dystrophic pathology and obvious cardiac symptoms, except high serum CK levels and weak signals of dystrophin expression as represented by immunohistochemistry. This striking finding can be further supported by another case of an elder patient with an exons 3–9 deletion; he was asymptomatic and active without any muscle involvements until the age of 67 years when the molecular diagnosis was made with both PCR and Southern blotting [41]. Although only two clinical reports are available so far, the LOVD has enrolled 10 patients carrying the deletion of exons 3–9 as determined by MLPA. Of them, phenotypes have been provided to six patients, of which 83% (5 out of 6) have been diagnosed with BMD and the remaining one diagnosed with DMD (the other 4 to be determined).

**Figure 7:**
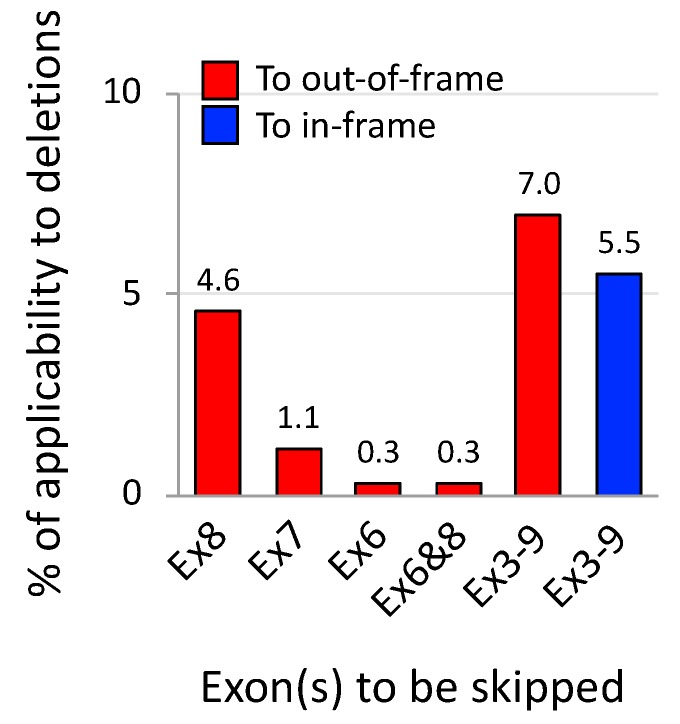
Theoretical applicability of exons 3–9 skipping to patients with out-of- and in-frame deletions. From the Leiden DMD database, 3232 patients with out-of-frame deletions and 1611 with in-frame deletions were extracted and were calculated for the applicability of certain exon skipping strategies to out-of- (red) and in-frame deletions (blue), respectively. Exons 3–9 skipping might become a potential therapy for patients with deletions in the proximal hot spot region that cause more variable severity.

Being an emerging therapeutic concept, AO-mediated exons 3–9 skipping is, therefore waiting for pre-clinical evidence. Encouragingly, for testing the proof-of-concept, appropriate animal models are available such as dystrophic dog models, called the Canine X-linked Muscular Dystrophy in Japan (CXMD_J_) and Golden Retriever Muscular Dystrophy (GRMD) [76]. These dystrophin-deficient dog models have an acceptor splice site mutation in intron 6 of the *dystrophin* gene and produce exon 7-deleted out-of-frame mRNA which is amenable to exons 3–9 skipping. Importantly, these dog models show variable severity even among littermates due to different expression levels of modifier genes such as phosphatidylinositol transfer protein-α (PITPNA) [77], which is as observed among patients with a deletion of exons 3–7 [76]. The experimental advantages of the dog models have been confirmed in the testing of exons 6 and 8 skipping with AO cocktails that is applicable to approx. 0.3% of out-of-deletions; its technical practicability has been demonstrated with the functional recovery of skeletal or cardiac muscles, accompanied by dystrophin rescue [78,79] (see Table 2 for research achievements using the dog models). The studies mentioned have provided clear orientation for examining the therapeutic potential of exons 3–9 skipping. This strategy can be theoretically applied to a higher 7% of out-of-frame deletions (226 in 3232) compared to single exon skipping approaches (Figure 7). Phenotypic variations are more frequently observed with deletions in the 5’ hot spots, including in-frame deletions. Exons 3–9 skipping, with its potential to induce functional dystrophin production, may become a therapeutic option for patients with unstable dystrophin derived from unfavorable in-frame transcripts; for example, exon 5-deleted mRNA [73]. Such applicable cases reach approx. 6% of in-frame deletions (89 in 1611) in theory.

As for the function of ABD1-affected dystrophins, Olson and colleagues have recently reported that exons 3–9-deleted dystrophin has more functional relevance compared to other dystrophin isoforms produced by exons 6–9 or exons 7–11-deleted transcripts [75] (Table 2). In the study, they generated three lines of hiPSC-derived cardiomyocytes with in-frame deletions of exons 3–9, 6–9, or 7–11 using CRISPR/Cas9 genome editing; they also engineered cardiac muscles using these cells for further functional testing. In the cell models, levels of rescued dystrophin and functional recovery with spontaneous Ca^2+^ activity and contractile performance were examined. Interestingly, cardiomyocytes expressing exons 3–9-deleted dystrophin showed greater expression of dystrophin and the most efficient function in terms of Ca^2+^ transient kinetics. The functionality of exons 3–9-deleted dystrophin was further confirmed in DMD iPSC-derived cardiomyocytes that have an out-of-frame deletion of exons 7–8. This in vitro functional assessment provides the rationale to move the concept of exons 3–9 skipping forward in vivo. Further studies are required to examine the therapeutic effects of exons 3–9 skipped dystrophin in skeletal and cardiac muscles using animal models such as the dog models described above. Along with functionality, the clinical relevance of obtained expression levels of rescued dystrophin as a result of treatment need to be clarified more with in vivo research. While ~15% of normal dystrophin amounts has been reported to correlate with an asymptomatic course in a patient with exons 3–9 deletion [40], other studies have reported that patients with the same deletion yielded the dystrophin levels comparable with normal levels [66] or approx. 47% of normal levels [41]. Because the number of patients we can refer to having the deletion is limited, more information with severity and dystrophin levels is desired for estimating the therapeutic benefit of exons 3–9 skipping.

## 6. Chemical Modification of AOs for Enhancing the Ability to Skip Multiple Exons

A major advantage of AO-mediated exon skipping is that its therapeutic effects can be drastically altered depending on the chemical structures of AOs used for the approach, along with optimization of AO sequences designed (reviewed in the next section). Over the last two decades, a variety of chemistries have been developed for increasing the potency of therapeutic AOs via modifications of the sugars, phosphate backbones and derivatives part of or conjugated to AOs (see the review [88]). These chemical modifications are utilized to enhance cellular uptake, binding affinity to target RNAs and nuclease resistance while minimizing adverse side effects such as immune stimulation and hepatic/renal toxicity. Using these modifications in combination, several AO compounds towards clinical translation of *DMD* exon skipping have emerged and advanced such as 2′-*O*MePS [89,90], phosphorodiamidate morpholino oligomers (PMOs or morpholinos) [78,91], vivo-PMOs [42,57,84], cell-penetrating peptide (CPP)-conjugated PMOs (PPMOs) [79,92], peptide nucleic acids (PNAs) [93], locked nucleic acids (LNAs) [94] and tricyclo-DNAs (tcDNAs) [95]. They have been tested in a number of pre-clinical trials with single or multiple exon skipping [96]. Of them, PMOs are the most promising chemical compound in terms of efficacy and safety, as is evident from the fact that a PMO-based AO has been conditionally approved by the FDA as the first exon skipping drug targeting *DMD* exon 51 [29] and that subsequent PMO drug candidates for skipping exon 45 or 53 are being tested in clinical trials [30,31]. Through pre-clinical and clinical experiences, however, major drawbacks of PMOs have been recognized: lower efficiency at skipping exons in skeletal muscles of intravenously-treated patients than that expected in animal studies [29], little efficacy in the heart [78,97] and rapid clearance from muscles [98]. This low bioavailability could be related to a limitation of the guanine content of PMO sequences that regulates the binding affinity to target RNA sequences [99] and difficulty in synthesizing the phosphorodiamidate backbone with pure stereochemical configuration that has the potential to enhance the ability of PMOs [100], as demonstrated in a study with AOs having phosphorothioate stereochemistry [101]. These issues, therefore, hinder the therapeutic development of PMO-mediated multiple exon skipping.

### 6.1. Vivo-PMOs

In this context, vivo-PMOs have been successful for use in multiple exon skipping in dystrophic mouse and dog models. A vivo-PMO is characterized by the conjugation of a PMO to an octa-guanidine dendrimer that enables the efficient cellular delivery of PMOs [102]. With this chemical property, the feasibility of in vivo exons 45–55 skipping has been demonstrated; systemic injections of a 10-vivo-PMO cocktail excluded 10 different exons simultaneously from a pre-mRNA in skeletal muscles of *mdx52* mice harboring an exon 52 deletion [42]. Another case of success is with exons 6 and 8 skipping using a 4-vivo-PMO cocktail in a dystrophic dog model (CXMD_J_) where *dystrophin* exon 7 is excluded from mRNAs [84]. In this case, intramuscular injection of the 4-vivo-PMOs at the lower dose of 0.4 mg each led to greater efficiency at skipping exons 6–8 and rescuing dystrophin compared to 1.2 mg each of cocktail 4-unmodified PMOs. Regarding toxicity, while no obvious adverse effects were found in blood tests in mice after long-term systemic treatment with cocktail 10-vivo-PMOs [57], a different study in mice has shown that chances of blood clotting and lethal ischemia can increase due to the cationic charge of the dendrimer moiety and the dimerization of vivo-PMOs [103]. An implication from this report is that toxicity derived from chemical characteristics needs to be carefully examined in developing cocktails composed of different AOs because the amounts of conjugates and chemically-modified nucleotides will be higher in cocktail AOs than that when using only one AO.

### 6.2. Cell-Penetrating Peptide-Conjugated PMOs

Although some issues have been raised, PMO-based AOs have advantages of steady supply, high solubility, charge neutrality (relating to its safety and application) and structural stability [96,104]. Given such chemical properties, much effort has been made with the addition of cell-penetrating peptides, CPPs to PMOs [92,105]. CPPs are principally composed of arginine-rich amino-acid residues for improving the low bioavailability of PMOs in vivo, which enables the administration of lower dosage of PMOs in treatment [106]. Thus, in *DMD* exon skipping, CPP-conjugated PMOs—called PPMOs—are widely tested in pre-clinical trials as a potential therapeutic agent to enhance exon skipping and dystrophin rescue in bodywide muscles including the heart [79,107]. A PPMO drug candidate for skipping human *DMD* exon 50 (AVI-5038, AVI BioPharma [now Sarepta Therapeutics]) was previously evaluated in healthy non-human primates and showed certain levels of skipping in skeletal and cardiac muscles [106]. Although AVI-5038 has been dropped as a drug candidate due to renal toxicity found in this pre-clinical study, using a different CPP composition another PPMO for *DMD* exon 51 skipping (SRP-5051, Sarepta Therapeutics) is currently being tested in a clinical trial (phase 1/2a, ClinicalTrials.gov ID: NCT03375255) and the FDA has accepted the Investigational New Drug application for this candidate [87].

The efficacy of PPMOs can be linked to their longer persistence in and more efficient distribution to tissues compared to PMOs [108,109]. Recently, the feasibility of PPMO-mediated multiple exon skipping therapy has been proven in our study [79]; exons 6–8 skipping with a 3-PPMO cocktail led to the expression of dystrophin in the heart of dystrophin-deficient dogs and restored the cardiac conduction abnormality in these dogs without detectable toxicity. Also, this study was the first to show that 3-different PPMOs can be evenly distributed among the various types/portions of skeletal and cardiac muscles. The long-term persistence of 3 PPMOs (4 mg/kg/each) has been confirmed in the muscles and blood at certain levels over 2 weeks or more after the intravenous injection [79], while the blood levels of 4-unmodified PMOs intravenously injected at 50 mg/kg/each were reduced in a shorter time of 1 week or less in dystrophic dog neonates [87]. In this study, four i.v. injections of 3-PPMOs (12 mg/kg in total, 2-week intervals) induced no obvious toxicity in dystrophic dogs, similarly to a previous study with *mdx* mice [110]. However, renal tubular degeneration has been reported in healthy cynomolgus monkeys intravenously injected with a PPMO compound at 9 mg/kg once weekly for four weeks [106]. Although further examinations regarding adverse effects are needed, it seems that PPMOs can become an ideal chemistry used for multiple exon skipping therapy to restore dystrophin expression in both skeletal and cardiac muscles.

It should also be mentioned that PPMOs have the potential for solving one of the more challenging tasks in *DMD* exon skipping; that is, how AOs can be specifically and selectively delivered into target muscles. In gene therapy, using different serotypes of adeno-associated viruses that have tropism for different tissues may become a solution [111]. In the antisense field, though some tissue tropisms are observed depending on chemical properties [112], muscle-specific delivery has not been achieved yet. The cell/tissue-selective targeting of AOs may be feasible using CPPs, as the transportation mechanism of PPMOs into cells could be controlled in response to the conformational differences among CPPs. Indeed, Kumar et al. have demonstrated the selective targeting for neurons using small interfering RNA (siRNA) binding to a CPP composed of a part of the rabies virus glycoprotein with neurotropic nature [113,114]. To increase the selectivity of cell-type specific delivery, other approaches with CPP design have been made mostly for siRNAs [92]. The sufficient accumulation of different AOs in a target cell is an essential process to achieve successful skipping of multiple exons. The development of CPPs that enable the selective introduction of combinational AOs into DMD muscles should serve to enhance the efficacy and safety of multiple exon skipping therapy.

## 7. Design of Antisense Sequences and Cocktails for Multiple Exon Skipping

### 7.1. Screening and Optimization of Individual Antisense Oligonucleotide Sequences

The design of antisense oligonucleotide (AO) sequences and the selection of target positions in pre-mRNA are key components to determine the success of exon skipping [91,115]. This insists upon the rigorous optimization of AO sequences with a valid screening system using a computational approach followed by empirical approaches, that is, in vitro and in vivo tests. In in silico pre-screening, a number of AO sequences designed for a given exon will be comprehensively evaluated to provide a reasonable selection of potentially effective ones to be tested in vitro. Subsequent cell-based screening confirms the actual ability of the selected AOs to skip a target exon, which allows for narrowing down the list of potential drug candidates. Finally, in vivo efficacy of a few AOs that show greater effectiveness in patient cells is tested in an appropriate “humanized” animal model (discussed in the next section). These processes are an example of a scheme to identify an AO drug candidate to be tested in clinical trials. Multiple exon skipping is achieved by a number of AOs targeting different exons. The efficacy of multiple exon skipping using combinational AOs as a cocktail can largely depend on the ability of individual AOs to skip an assigned exon [81,84]. Accordingly, the drug-discovery effort will be directed at optimizing the sequence of respective AOs composing a cocktail, which can be a reason to make the development of multiple exon skipping therapy difficult.

In an AO optimization process, target pre-mRNA positions are mostly assigned to exons rather than introns because of more unique/specific sequences of exons. Besides exon sequences, AO length is one of the more important design criteria optimized in response to the selected chemistry used, for example, 13–18-mer for LNA-based AOs [116,117], 18–20-mer for 2′-*O*MePS-AOs [118] and 21–30-mer for PMOs [31,91]. According to such lengths, a few hundred AO sequences can be designed for a single target exon. However, in practice, only a restricted number of candidate AOs usually proceed to subsequent in vitro screening, selected based on parameters thought to relate to the activity of AOs; actually, such selection can involve looking at more than sixty parameters (see [115] for details). Previously, a major drawback was that these parameters were not integrated into a single evaluation value for the prediction of potentially effective sequences, making the optimization process complicated.

To overcome this limitation, our group has previously consolidated important parameters with great predictive power and established an algorithm to provide a predicted exon skipping efficiency value as the consolidated single output measure [115]. Although currently limited to PMO-based AOs, the in silico predictive tool enables the rational design of AO sequences for any exon of the *DMD* gene. Indeed, using this computational approach followed by empirical testing, we have already succeeded in identifying effective PMO sequences for skipping exons 44, 51, or 53 [91,115]. In our studies, it was revealed that the efficiency of exon skipping could be altered by more than 20-fold upon the use of more optimal PMO sequences. Surprisingly, most of our newly designed PMOs for exon 51 skipping showed significantly higher efficiencies in vitro compared to the analog of the first FDA-approved PMO drug, eteplirsen, and we have demonstrated significant in vivo exon skipping efficacy of our best PMO with a humanized mouse model that has the human *DMD* gene, indicating that the PMO we found could become the next drug candidate for DMD therapy [91]. This screening model can be applied to the evaluation of AOs targeting other *DMD* exons, which would facilitate the development of cocktail drugs composed of only AOs that have a reliable skipping effect on the assigned exon.

### 7.2. Antisense Oligonucleotide Cocktails Tailored to Patient Mutations

In this context, exons 45–55 skipping is expected to be achieved by tailored combinations of AOs to mutation patterns. In order to treat all the mutation patterns arising within the exons 45–55 region, at least eleven effective AOs, each targeting respective exons in the region, need to be developed. Once such AOs are prepared, a cocktail drug can be formulated with only the required AOs to induce exons 45–55 skipping; for example, 10-different AOs to skip exons 46–55 are mixed as a cocktail drug for treating the most frequent exon 45 deletion and a single type AO can be used for other common deletions of exons 45–54 and exons 46–55 (Figure 2). Indeed, we have shown the possibility of this tailored combination approach using different cocktails of PMO-based AOs in DMD-patient derived myotubes carrying the deletions of exons 45–50 and 46–50 [58]. In this study, we designed cocktail AOs while considering the prevention of dimerization between different AO sequences which could reduce antisense activity and become a cause of potential side effects [99,103,119]. The potential formation of self- and hetero-AO dimers in a cocktail can be calculated as the Gibbs free energy (dG) of binding between AOs using prediction tools for nucleotide secondary structures such as RNAstructure [120], which could serve as a means to avoid duplex formation. For treatment, mixing different AOs just before use is pragmatically crucial in preventing AO dimerization and enabling the appropriate evaluation of the efficacy and safety of AO cocktails. To establish this concept, further investigations are required such as whether possible combinations can produce expected results associated with rescued dystrophin expression.

In exons 45–55 and 3–9 skipping that aim to induce the production of functionally-corrected dystrophin, it is clear that mutation-tailored AO cocktails, that is, compositions of only AOs assigned to existing exons in the gene of patients are preferable in terms of efficacy and safety rather than the administration of all AOs including ones targeting absent exons in the gene. However, the development of such AO cocktail drugs faces significant hurdles with the current drug regulatory system, as well as with future clinical trial design. Regulatory authorities such as the FDA and the European Medicines Agency (EMA) have not accepted cocktail AOs to be one drug and its use in combination because they consider individual AOs to be separate drugs according to the nature of AOs working in a sequence-specific manner [49]. In the development of AO cocktail drugs based on this regulation, safety testing of each AO in a cocktail are required followed by that of possible combinations composed of approved AOs. To cover all patients amenable to exons 45–55 skipping approaches, at least eleven-different AOs targeting individual exons within the exons 45–55 region need to be approved as medical products. The theoretical number of AO combinations can reach as many as the number of existing patient mutation patterns; 36 patterns of out-of-frame deletions are correctable with this strategy in the LOVD registration (as of 22 June 2018). This combinational number in cocktails can further increase when DMD patients having in-frame deletions and other mutation types come to medical attention. It is important to note that clinical trials need to be set for individual cocktail compositions in the current situation. However, in practice, clinical trials of all of those AO combinations are difficult in terms of recruiting sufficient patients including those for placebo controls [121]. A potential solution might be that AOs approved as a cocktail drug through a well-designed clinical trial are used as different combinational cocktails; for example, in case if a 10-AO cocktail for an exon 45 deletion, the most common DMD-related deletion (Figure 2) were approved, appropriate 5 and 3 AOs out of the 10 AOs would be adapted as cocktail drugs to those with the deletions of exons 45–50 and 45–52, respectively. This might not be an unrealistic option because 5- and 3-AO cocktails are unlikely to lead to more side effects as far as administrated in the dose range defined in a clinical trial of 10-AO cocktail. Through careful assessment of various possibilities, existing regulatory approval processes may need to be modified toward clinical application of AO cocktail approaches in the future.

## 8. Patient-Derived Cells and Humanized Animal Models for Testing Multiple Exon Skipping

DMD patient-derived skeletal muscle cells and humanized dystrophic animals are indispensable tools for evaluating the efficacy and safety of exon-skipping AOs designed for patients. Although an AO drug candidate tested in patients should be determined by thorough testing with these models, the use of such models is limited. This section will discuss the challenges in DMD models for developing multiple exon skipping approaches that target human *DMD* transcripts.

### 8.1. Patient-Derived Muscle Cell Lines

Major issues in preparing DMD muscle cells include that: (1) DMD muscle cells with mutations of interest are not always available, (2) the ability of primary human myogenic cells to proliferate and differentiate to myotubes is limited and (3) the expression levels of *DMD* mRNA in primary DMD muscle cells and converted DMD muscle cells are usually low. Accordingly, these limitations pose problems when evaluating the efficacy of exon skipping with AOs designed for patients, particularly multiple exon skipping using many AOs. For these issues, there exist some solutions such as the use of immortalized DMD muscle cell lines [122,123], fibroblast-converted muscle cells [81,124], DMD-patient-derived iPSC lines that can differentiate to muscle cells [125,126,127,128], muscle cell lines with artificial mutations [129], or cells loading a *DMD* construct mediated-reporter system [130].

Of them, the immortalized DMD muscle cell lines can be the most powerful and convenient tool for research. Mouly and colleagues have created a large collection of immortalized human myoblasts derived from patients in a variety of neuromuscular disorders including DMD [122]. The immortalized cells that are generated by the *human telomerase* and *cyclin-dependent kinase 4*-expressing vectors, can be clonally expanded and easily enter the terminal differentiation process for myotubes. Importantly, the cell lines have been confirmed to retain the characteristics of primary muscle cells [131]. In this context, we employed immortalized DMD muscle cells to find an AO drug candidate that deserves to move forward into clinical trial testing [91]. The immortalized DMD cells allowed for statistically comparing the levels of exon skipping and rescued dystrophin induced by designed AOs. Through the study, we have found a novel exon 51 skipping AO with significantly greater effectiveness than the FDA-approved one (eteplirsen). This methodology can be applied to the development of cocktail AOs used for multiple exon skipping. AOs comprising a cocktail must be individually optimized to skip each exon located in a target region. In AO screening, clinical drug candidates must be determined in a reproducible fashion, which is doable with immortalized DMD cell lines [91,115], DMD iPSC lines [125,126,128,132] or an exon skipping-reactive cell system [130].

For the limitation concerning available mutations for study, a genome editing system can provide a solution to create new cell lines with artificial mutations that mimic natural mutations. Such genetically engineered cells have been reported with DMD iPSCs and a rhabdomyosarcoma cell line that are amenable to skipping exons 45–55 [59,129] and exons 3–9 [75]. In order to develop multiple exon skipping therapies, a variety of combination cocktails of AOs needs to be tested tailored to different deletion patterns. Continuing efforts to collect DMD cell lines with different mutations will be required [128].

### 8.2. Humanized Animal Models to Test Antisense Oligonucleotides Designed for Patients

A major hurdle in in vivo testing is that the efficacy of AOs designed for patients cannot be evaluated in dystrophic animal models because the sequence and splicing mechanism of the *dystrophin* genes in animals are different from those of the human *DMD* gene. Although anticipated, this problem was made obvious in our recent study; mouse version AOs that are equivalent to human AOs in terms of target position but with a few different bases changed, were tested in *mdx52* mice but could not produce effects as that found in DMD muscle cells treated with the human AOs [91]. Although animal models are helpful for confirming the concept of skipping exons of interest, our finding indicates that the mouse *Dmd* gene cannot be used for evaluating the skipping potential of RNA positions targeted by AOs designed for humans. To overcome this hurdle, a humanized mouse model, called the *hDMD* mouse model that has the normal human *DMD* gene but retains the mouse *Dmd* gene, was developed for testing in vivo efficacy of human AOs [133]. Although useful, in this humanized model it cannot be excluded that effects of tested human AOs may be confounded by the presence of the homologous mouse *Dmd* pre-mRNA. To prevent accidental binding to mouse pre-mRNA, a novel model has been generated; the *hDMD/Dmd-null* mouse that has human *DMD* but lacks the entire mouse *Dmd* [91]. Using this model, we have demonstrated that the exon 51 skipping efficiency of human AOs can be evaluated in the normal human *DMD* gene expressed in these mice [91]. Young et al. also utilized the *hDMD* model to create a “dystrophic” humanized mouse model in which *DMD* exon 45 was deleted by the CRISPR/Cas9 system to cause an out-of-frame mutation and human gene-derived dystrophin cannot be produced. The exon 45-deleted *hDMD* mice were crossed with dystrophin-deficient *mdx* or *mdxD2* mice that harbors a point mutation in their *Dmd* gene so that the *Dmd*-derived endogenous dystrophin cannot be expressed [60]. More recently, Veltrop et al. used the TALEN genome-editing system in an embryonic stem cell line of hDMD/*mdx* mice and created another version of the dystrophic humanized mouse model called del52hDMD/*mdx* that has an exon 52 deletion in the *DMD* gene [134]. This model produces no dystrophin due to mutations in both human and mouse genes. Putting together recent progress, an ideal model can be one holding *DMD* mutations and lacking the mouse *Dmd* gene. Although such a model is useful for developing *DMD* exon skipping therapies, it seems challenging to create a variety of humanized dystrophic models carrying mutations of interest, in particular, clustered mutations affecting multiple exons; for example, 36 models are theoretically required according to the number of cocktail combinations for exons 45–55 skipping against out-of-frame deletions arising within the exons 45–55 region. It should also be noted that the efficacy of human AOs found in humanized models is induced with animals’ splicing machinery. This fact strongly indicates that AO drug candidates targeting the human *DMD* gene need to be determined through a thorough evaluation with both human muscle cells, preferably primary cells closer to the nature of patients, as well as humanized animals.

## 9. Skipping of Exon Blocks Using Fewer Antisense Oligonucleotides

Although promising, a technical problem in AO-mediated multiple exon skipping is the difficulty in excluding all targeted exons from a pre-mRNA at the same time. In the current strategy, different AOs need to bind simultaneously to their respectively assigned exons in one pre-mRNA. To increase such a chance, the dosage of cocktail AOs will be increased. Accordingly, the current multiple exon skipping strategy faces issues of low skipping efficiency and high AO concentrations that can be associated with off-target effects. To address these issues, multiple exon skipping using fewer AOs, that is, skipping exon blocks could be a straightforward approach.

To develop such a strategy, further understanding of the RNA splicing mechanism is required [135]. Suzuki et al. have recently reported a possible mechanism by which endogenous exons 45–55 skipping occurs in the normal *DMD* gene [136]. The alternative splicing that leads to spontaneous multiple exon skipping is also found in the exons 2–18 region of the normal *DMD* gene [137], which corresponds to the hot spot at *DMD* 5’ side. The endogenous skipping of the entire region of *DMD* exons 3–9 has been reported in healthy individuals as circular RNAs generated by a back-splicing event [138]. Exon 3 [137] and exon 9 [81,139] are more likely to be skipped alternatively and endogenously as observed in the normal or mutated gene. Interestingly, a common feature among the introns flanking exons 3, 9, 45 and 55 (introns 2, 9, 44 and 55) is that the lengths are over 50 kb, which is exceptionally longer than most other introns [49]. It has been reported that in the normal *DMD* gene, the removal of longer introns (>10 kb) from pre-mRNA predominantly requires multi-step splicing processes such as recursive and nested splicing [140]. During the stepwise removal of introns, particular exons of which introns are spliced out in a single step are constitutively processed as “exon blocks” composed of two or more exons. Accordingly, the regions of exons 3–9 and exons 45–55 are divided into three exon blocks each: exons 3–5, 6–7 and 8–9 in the former and exons 45–49, exons 50–52 and exons 53–57 in the latter [140]. In the concerned introns, essential sites to skip the exon blocks and the variation of splicing machinery among mutation patterns remain to be fully elucidated. Furthermore, it is possible that different breakpoints in *DMD* introns, even though they generate the same exon deletion, may affect splicing patterns and reactivity to AOs, which needs to be carefully examined in exon skipping strategies. Revealing the inherent mechanisms behind these skipping events can provide insights into developing a therapeutic strategy that enables multiple exon skipping with only a few select AOs. It should also be noted that exon-block skipping using minimal AOs could lessen the possibility of side effects in cocktail therapies in which higher dosages are principally required for different AOs to act together on a pre-mRNA.

## 10. Conclusions

Owing to advances in diagnostic methods, global DMD databases are becoming more useful to uncover associations between *DMD* mutations and their severity in dystrophinopathy. These observations have provided direction on how the dystrophin structure can be modified so as to have preserved function resulting in therapeutic benefit. In this context, the feasibility of a multiple exon skipping approach, exons 45–55 skipping, has been demonstrated using AO cocktails accompanied by the recovery of muscle function in dystrophic animals. The emerging therapeutic concept of exons 3–9 skipping is also a prospective approach for treating patients with mutations in the proximal hot spot. AO cocktails for skipping multiple exons have, therefore, the potential for becoming a novel drug to produce functionally-corrected dystrophin. Along with the availability of promising AO modifications, the proper designing of effective AO sequences, an essential condition for the development of the cocktail drugs, is now doable using robust algorithms for predicting exon skipping efficacy. With advanced technologies such as cell immortalization and genome editing, appropriate DMD patient muscle cells and humanized animal models are now becoming more accessible for evaluating the therapeutic potential of designed AOs. In order to increase patient access to AO drugs, other challenges will also need to be further discussed, such as the current regulatory systems of AO-drug approval [141,142] and the high costs of AO therapy imposed on patients [143].

While the prevailing current AO-mediated exon skipping approach targets a single exon for treating DMD with out-of-frame deletion mutations, multiple exon skipping that aims for the functional correction of dystrophin can be applied to both out-of-frame and in-frame deletion mutations. Accordingly, more symptomatic patients with DMD or BMD are made theoretically treatable by this approach. Multiple exon skipping therapies are also needed for treating patients with other mutation types such as duplication and nonsense mutations that form large patient populations [22,144]. Indeed, there exist patients whose affected reading frame can be corrected only by excising multiple exons. In some cases of duplications, that are the second most common mutation type [22,144], reading frame restoration can be achieved by the skipping of additional exons as AOs supposedly skip both duplicated and native exons and consequently induce another type of out-of-frame mRNAs [32]. Nonsense mutations in exon 2, 6, 7, 8, 61, 67, 75, 76 and 78 can be rescued by the skipping of three or more exons [145]. Importantly, the applicability of multiple exon skipping approaches has been estimated to reach 80% and 98% of those with duplications [146] and nonsense mutations [145], respectively. With an increase in clinical and experimental evidence, the potential therapeutic opportunity of multiple exon skipping could be further expanded to patients even with rare mutations in the future. Towards its clinical application, systematic and comprehensive strategies in the development of multiple exon skipping is highly encouraged.

## Figures and Tables

**Table 1:** Pre-clinical studies with skipping or deleting the exons 45–55 region in the distal hot spot.

Strategy	Study Model	Mutation	Target Molecule	Carrier	Target Regions	Administration	Comments	Ref.
Skipping exons 45–55	Primary DMD myotubes	*DMD* ex46–50 or ex48–50 del.	pre-mRNA	2 or 12 2′-OMePSs	Ex45–55	PEI transfection	First published attempt at ex45–55 skipping in vitro using DMD patient cells	[56]
	*mdx52* mice	*Dmd* ex52 del.	pre-mRNA	10 vivo-PMOs	Ex45–51, ex53–55	i.m. (TA) or i.v.	First demonstration of successful in vivo ex45–55 skipping treatment in mice	[42]
	*mdx52* mice	*Dmd* ex52 del.	pre-mRNA	10 vivo-PMOs	Ex45–51, Ex53–55	i.m. (TA) or i.v.	Showed the long-term systemic efficacy and safety of ex45–55 skipping treatment in mice	[57]
	Converted DMD myotubes	*DMD* ex46–50 del.	pre-mRNA	6 PMOs	Ex45, Ex51–55	Endo-porter transfection	Demonstrated the feasibility of ex45–55 skipping in muscle cells transdifferentiated from patient fibroblasts and showed a possibility of tailored cocktail therapy	[58]
	Converted DMD myotubes	*DMD* ex45–50 del.	pre-mRNA	5 PMOs	Ex51–55	Endo-porter transfection		
Deleting exons 45–55	Immortalized DMD myotubes	*DMD* ex48–50 del.	DNA	CRISPR/Cas9	Introns44, 55	Plasmid electroporation: spCas9, 2 gRNAs	First study to successfully delete ex45–55 in vitro; NSG mice transplanted with treated myoblasts showed dystrophin-positive fibers	[59]
	DMD hiPSCs, hiPSC-derived myotubes, cardiomyocytes	*DMD* ex46–51 or 46–47 del., or ex50 dup.	DNA	CRISPR/Cas9	Introns 44, 55	Plasmid nucleofection: spCas9, 2 gRNA	Restored dystrophin expression/functionality in patient hiPSCs and derivative cell types	[61]
	DMD hiPSC-derived skeletal muscle cells	*DMD* ex46–51 del.	DNA	CRISPR/Cas9	Introns 44, 55	Engraftment into NSG-*mdx* mice TAs	Muscles engrafted with treated hiPSC muscle cells showed proper dystrophin and beta-dystroglycan localization	
	hDMD del. 45 mice	*DMD* ex45 del.	DNA	CRISPR/Cas9	Introns 44, 55	Electroporation into FDB muscle	First to show dystrophin restoration in vivo in humanized dystrophic mice following ex45–55 deletion, without cell transplantation	[60]

ex, exon; del., deletion; 2′-OMePS, 2′-O-methyl-phosphorothioate; PEI, polyethyleneimine; PMO, phosphorodiamidate morpholino oligomer; i.m., intramuscular injection; i.v., intravenous injection; del, deletion; dup, duplication; TA, tibialis anterior muscle; CRISPR/Cas9, clustered regularly interspaced short palindromic repeats (CRISPR)/CRISPR-associated nuclease (Cas) 9; spCas9, *Streptococcus pyogenes* Cas9; gRNA, a short guide RNA; hiPSCs, human induced pluripotent stem cell; NSG, NOD *scid* IL2R gamma; hDMD, a mouse model with the human *DMD* gene; FDB, flexor digitorum brevis muscle.

**Table 2:** Pre-clinical studies with skipping exons 6 and 8 and deleting exons 3–9 in the proximal hot spot.

Strategy	Study Model	Mutation	Target Molecule	Carrier	Target Regions	Administration	Comments	Ref.
Skipping exons 6 & 8	GRMD dog myotubes	An ASS point mutation in *dystrophin* intron 6 that results in ex7 removal from mRNA	pre-mRNA	2 2′OMePSs/PMOs/PPMOs	Ex6, intron 7	lipofectamine (2′-OMePS) or no agent(PMO/PPMO)	Showed the potential of using a canine DMD model for testing multiple exon skipping therapies	[80]
	CXMD_J_ dogs	An ASS mutation in *dystrophin* intron 6	pre-mRNA	3 PMOs	Ex6, 8	i.m. (TA, ECU) or i.v.	First published in vivo exon skipping study using a canine DMD model	[78]
	Converted myotubes of DMD patient or CXMD_J_	*DMD* ex7 del. (patient), an ASS mutation in *dystrophin* intron 6 (dog)	pre-mRNA	3 or 4 PMOs	Ex6, 8	endo-porter transfection	Demonstrated the feasibility of adapting the canine ex6-8 skipping strategy into patients	[81]
	GRMD dogs	An ASS mutation in *dystrophin* intron 6	pre-mRNA	rAAV6-U7 snRNA construct	Ex6, 8	transendocardial injection	Reported the long-term efficacy of ex6-8 skipping for rescuing dystrophin and improving cardiac function in vivo	[82]
	GRMD dogs	An ASS mutation in *dystrophin* intron 6	pre-mRNA	rAAV6-U7 snRNA construct	Ex6, 8	i.c. or transendocardial injection	Improved transendocardial delivery of ex6-8 skipping snRNAs into the heart using MRI-based injection guidance	[83]
	CXMD_J_ dogs	An ASS mutation in *dystrophin* intron 6	pre-mRNA	4 vivo-PMOs	Ex6, 8	i.m. (forelimb muscles)	First study to show in vivo dystrophin rescue in a canine DMD model using modified/chemically-conjugated PMOs	[84]
	GRMD dogs	An ASS mutation in *dystrophin* intron 6	pre-mRNA	rAAV1-U7 snRNA construct	Ex6, 8	i.m. or transvenous perfusion (forelimb muscles)	Demonstrated improvements in muscular strength following ex6-8 skipping	[85]
	GRMD dogs	An ASS mutation in *dystrophin* intron 6	pre-mRNA	rAAV8-U7 snRNA construct	Ex6, 8	transvenous forelimb perfusion	Further supported use of locoregional delivery for rAAV-packaged ex6-8 skipping snRNA therapy in patients	[86]
	CXMD_J_ dogs	An ASS mutation in *dystrophin* intron 6	pre-mRNA	3 PPMOs	Ex6, 8	i.m. (TA), i.c. or i.v.	Demonstrated the therapeutic utility of PPMO-based exon skipping for ameliorating cardiac conduction defects in vivo	[79]
	CXMD_J_ dogs	An ASS mutation in *dystrophin* intron 6	pre-mRNA	4 PMOs	Ex6, 8	i.v.	Reported the efficacy of ex6&8 skipping therapy in dystrophic dog neonates, highlighting the need for earlier treatment for a dystrophic pathology	[87]
Deleting exons 3–9	DMD hiPSCs, hiPSC-derived cardiomyocytes	*DMD* exons 8–9 del. (induced/native)	DNA	CRISPR/Cas9	Introns 2, 7	plasmid nucleofection: spCas9, 2 gRNA	First and only ex3-9 deletion study to date; more functional dystrophin from ex3-9 deleted mRNA than ex6-7 or 7-11-deleted dystrophin; improved cardiomyocyte calcium-handling functions upon treatment	[75]

GRMD, Golden Retriever Muscular Dystrophy; CXMD_J_, beagle-based Canine X-linked Muscular Dystrophy in Japan (CXMD_J_); ex, exon; ASS, acceptor splice site; PMO, phosphorodiamidate morpholino oligomer; PPMO, peptide-conjugated PMO; i.c., intracoronary artery injection; AAV, adeno-associated virus; snRNA, small nuclear RNA.
